# Robust Prediction-Reliability Assessment in Biomedical Sensing and Classification Under Data Scarcity Using Conformal Prediction and Statistical Randomization

**DOI:** 10.3390/s26206513

**Published:** 2026-10-14

**Authors:** Yuqi Zhou, Xianghao Zhan

**Affiliations:** 1 Department of Statistics, Zhejiang University, Hangzhou 310058, China; zhouyuqi@zju.edu.cn; 2 Department of Biomedical Data Science, Stanford University, Stanford, CA 94305, USA

**Keywords:** biomedical sensing, sensor data, biomedical AI, prediction reliability, conformal prediction, statistical randomization, data scarcity, electronic nose

## Abstract

Reliable assessment of prediction reliability is important in biomedical sensing and measurement applications, where machine learning models increasingly transform imaging, physiological, chemical, and molecular measurements into clinically relevant predictions. Such applications often operate under limited labeled data because sensor acquisition, experimental measurement, and expert annotation can be costly, while biological and measurement variability can further challenge model development. Under these conditions, reliability scores derived from standard inductive conformal prediction (ICP) can be sensitive to the particular division of available data into proper-training and calibration subsets. To address this issue, we propose three randomized ICP variants: bootstrapping ICP (BICP), randomized ICP (RICP), and out-of-bag ICP (OOB-ICP), to improve the informativeness and repeated-sampling stability of conformal-derived prediction-reliability scores under data scarcity. We benchmarked these methods using a controlled simulation and three real-world biomedical sensing and measurement applications: pneumonia diagnosis from X-ray radiography, alternative herbal medicine classification using a 16-channel semiconductor electronic-nose gas-sensing system, and breast cancer subtyping from RNA-seq molecular measurements. Results show that the proposed methods produce aggregate credibility scores that are informative of prediction correctness and less sensitive to randomness in training-data sampling. Empirical prediction-set coverage analyses further characterize the behavior of the aggregated conformity scores relative to nominal error levels without assuming that they retain the exact finite-sample validity of conventional conformal *p*-values. Among the evaluated methods, OOB-ICP achieved strong overall classification performance and informative, stable prediction-reliability scores, supporting statistical randomization as a practical strategy for stabilizing conformal-derived reliability assessment under limited training data in biomedical sensing and measurement applications.

## 1. Introduction

Machine learning (ML) has become an important component of modern biomedical sensing and measurement systems, enabling complex sensor- and assay-derived signals to be transformed into clinically or biologically meaningful predictions. Representative applications span medical imaging [1], physiological and wearable health sensing [2,3], electronic-nose and chemical sensing [4,5], and high-throughput molecular measurements [6,7]. These sensing-driven AI pipelines frequently operate under practical constraints including limited labeled samples, heterogeneous biological signals, measurement variability, and costly expert annotation. In such settings, predictive accuracy alone may be insufficient, particularly when model outputs are used to support high-stakes biomedical decisions. Complementary information regarding the reliability of individual predictions can help researchers and practitioners interpret model outputs and identify cases that may warrant additional measurement or human review [8,9,10].

Conformal prediction (CP) provides a rigorous statistical framework for constructing prediction sets with distribution-free marginal coverage guarantees under appropriate exchangeability assumptions [11,12,13,14,15]. In classification settings, conventional conformal procedures additionally yield label-wise conformal *p*-values, from which quantities such as credibility and confidence can be derived to characterize prediction reliability. These quantities have been used to provide case-level prediction-reliability information beyond the predicted label in biomedical classification applications, including electronic-nose-based herbal medicine quality control [4] and lung cancer diagnosis based on breath air [5], silent speech/taste sensation recognition based on surface electromyography [16,17], where confidence and credibility provide case-level information beyond the predicted label. More recently, inductive CP (ICP) has also been leveraged in biomedical machine learning pipelines for noisy training data cleaning across natural language processing, imaging, electronic health records, and RNA-sequencing tasks [18], highlighting its broader benefits in robust ML modeling under noisy real-world biomedical datasets. These prior studies demonstrate the relevance of CP to sensor-driven biomedical AI, where model predictions derived from noisy and heterogeneous measurements may benefit from accompanying case-level reliability information.

Despite its merits and applicability, the practical use of CP in biomedical sensing applications faces important challenges under limited data. Sensor- and measurement-derived biomedical datasets may be difficult or expensive to acquire and label at scale because data collection can require specialized instrumentation, controlled experimental procedures, patient recruitment, or expert annotation [19,20]. Consequently, the number of labeled observations available for both model fitting and conformal calibration may be limited. In addition, biological heterogeneity and measurement variability can further increase sensitivity to the particular samples available for model development. Second, standard ICP divides the available labeled data into a proper-training set for model fitting and a calibration set for computing calibration nonconformity scores. Although this separation is fundamental to the ICP procedure, it reduces the effective sample size available at each stage. Under limited-data conditions, reliance on a single proper-training/calibration partition can also introduce substantial partition-induced variability: different realizations of the available training data and different assignments of samples to the proper-training and calibration subsets can change both the fitted classifier and the resulting conformal credibility scores. Consequently, the prediction-reliability assessment obtained from a single ICP partition may be sensitive to finite-sample and partition randomness. This sensitivity is especially relevant in biomedical AI, where prediction-reliability assessment should remain informative and stable even when labeled data are scarce, noisy, or affected by sampling variability [9].

To address these challenges, we investigate robust randomized ICP methods that aim to improve the stability of prediction-reliability assessment in biomedical AI under training data scarcity. The present study focuses on conformal classification under limited labeled data. Specifically, we propose and systematically benchmark three approaches: bootstrapping ICP (BICP), randomized ICP (RICP), and out-of-bag ICP (OOB-ICP). These methods leverage statistical resampling and repeated calibration-partition strategies to mitigate the sensitivity of prediction-reliability assessment to limited training data. For the proposed resampling-based methods, conformal *p*-values obtained from individual resampling iterations are aggregated across repetitions, and the resulting quantities are interpreted as aggregate conformity scores rather than exact conformal *p*-values. The corresponding maximum score across candidate labels is therefore referred to as the aggregate credibility score, which is evaluated as a prediction-reliability measure. Accordingly, the proposed methods are intended to reduce finite-sample and partition-induced variability in conformal-derived prediction-reliability scores; they are not presented as new formally calibrated uncertainty measures. We systematically benchmark the proposed methods across multiple biomedical modalities in terms of predictive performance, reliability discrimination, score stability, and empirical prediction-set coverage.

From a sensing perspective, the methodological objective of this study is therefore to provide more stable prediction-reliability assessment when machine learning models are trained on limited biomedical sensing and measurement data. We evaluate this objective using complementary modalities spanning X-ray imaging, semiconductor electronic-nose gas sensing, and RNA-seq molecular measurements, together with a controlled simulation. These applications differ substantially in sensing principle, signal representation, dimensionality, and sample availability, allowing us to examine whether the proposed reliability framework generalizes across heterogeneous biomedical measurement pipelines. The electronic-nose task provides a direct multichannel sensor-system application, whereas the imaging and transcriptomic tasks extend the evaluation to complementary biomedical sensing and measurement modalities.

## 2. Materials and Methods

### 2.1. Datasets

To evaluate the proposed framework across heterogeneous biomedical sensing and measurement settings, we considered one controlled simulation and three real-world applications spanning medical imaging, volatile-organic-compound gas sensing, and molecular transcriptomic measurement (Table 1). These modalities represent substantially different measurement processes and feature spaces, enabling evaluation of prediction-reliability stability across complementary sensing-driven biomedical AI tasks.

**Simulated binary classification:** The simulation dataset was designed as a controlled two-dimensional binary classification task to evaluate model behavior under different levels of data scarcity in a controlled manner. We generated a total of 6000 observations from two overlapping bi-variate Gaussian distributions. Class 0 was sampled from a normal distribution with mean vector (0.5,0.5), and class 1 was sampled from a normal distribution with a mean vector (−0.5,−0.5). Both classes shared the covariance matrix

$$\Sigma =\left(\begin{array}{cc} 1.5 & 0.3\\ 0.3 & 1.5 \end{array}\right).$$

A stratified 80%/20% split was first performed to split the fixed test set, and 10 independent random training-set samplings were performed to test model robustness, with 4800 samples forming the available training pool and 1200 samples retained as a fixed test set. The fixed test set was kept unchanged across all repeated experiments and all evaluated training-set sizes.

**Pediatric pneumonia diagnosis from X-ray radiography:** The pediatric pneumonia diagnosis task was based on PneumoniaMNIST [1], a subset of the MedMNIST collection. It contains 5856 grayscale chest X-ray images with a resolution of 28×28 pixels, labeled as pneumonia or normal. Image-level feature representations were extracted using the image encoder of BiomedCLIP [21], a biomedical vision–language foundation model. As a radiographic sensing modality, chest X-ray imaging converts tissue-dependent X-ray attenuation into spatial image measurements, providing a complementary sensor-derived biomedical data type to the multichannel chemical-sensing signals evaluated in the electronic-nose task. These BiomedCLIP embeddings were used as input features for downstream classification and conformal prediction; feature standardization was performed within the model-fitting pipeline as described in the next section (proper-training set) rather than globally before data splitting. We followed the released PneumoniaMNIST data partitions: the released training and validation partitions were combined to form the available training pool, whereas the released test partition was retained as a fixed held-out test set throughout all 10 repeated experiments and training-set-size conditions. The released test set was not used for model fitting, calibration, preprocessing fitting, or resampling.

**Alternative herbal medicine classification using electronic-nose sensing**: This task provides a direct multichannel sensor-system application of the proposed reliability framework. The dataset was acquired using an electronic-nose system comprising 16 semiconductor-based gas sensors with differential sensitivity to volatile organic compounds (VOCs) [4,22]. This dataset was collected to classify 12 alternative medicine classes (Astragalus, Liquorice, Chinese Angelica, Saposhnikovia Divaricata, Radix Angelicae Pubescentis, Radix Angelicae Dahuricae, Notopterygium Incisum, Codonopsis Pilosula, Radix Bupleuri, Ligusticum Chuanxiong Hort, Radix Peucedani, and Pueraria Lobata), with 50 samples per class and 600 samples in total. After preprocessing and feature extraction, each sample was represented by a 128-dimensional electronic-nose signal vector including the maximum, integral, phase and exponential moving average of derivatives for each channel of the 16 semiconductor voltage (Figaro Engineering Inc., Osaka, Japan) response [4]. This dataset was used as a real-world small-sample multi-class gas-sensing task. For each train/test partition within 10 parallel experiments, stratified sampling was performed within each class: 10 samples per class were held out as the test set, while the remaining 40 samples per class formed the available training pool. Thus, each train/test split contained 120 test samples and 480 training-pool samples, with no overlap between the two sets.

**Breast cancer subtyping from molecular measurements**: The breast cancer subtyping task was based on TCGA RNA-sequencing data [7,18], representing a high-dimensional molecular measurement modality complementary to the imaging and gas-sensing applications. The dataset contains 1096 samples before filtering, with five molecular subtype labels: LumA, LumB, Basal, HER2, and normal-like. To reduce the instability and simplify the challenge of this task caused by the relatively fewer minority-class samples and severe class imbalance, the final analysis retained the three most frequent molecular subtypes (LumA (*n* = 555), LumB (*n* = 210), Basal (*n* = 184)). The resulting RNA-seq input matrix contained 31,098 gene-expression features. A log(1+x) transformation was applied to the raw counts. Similar to previous datasets, we performed 10 repeated stratified outer train-test splits, with 75% of the samples assigned to the available training pool and 25% retained as held-out test data in each repetition. Further preprocessing and feature selection were performed within the model-fitting pipeline using proper-training data only, as described in the next section.

### 2.2. Robust Conformal Prediction Methods

Inductive conformal prediction (ICP) is a computationally efficient conformal framework that uses a proper-training set to fit the predictive model and a separate calibration set to construct label-wise conformal *p*-values and prediction sets. Under the standard exchangeability assumptions, conventional ICP provides finite-sample marginal coverage guarantees for the resulting prediction sets [13,16,18]. In classification, the conformal *p*-values can also be summarized through quantities such as credibility to provide sample-level prediction-reliability information. To be specific, in standard ICP, the labeled training data are split into a proper-training set and a calibration set:

$$D_{\mathrm{proper}}={\left\{\left(x_{i},y_{i}\right)\right\}}_{i=1}^{m},\;D_{\mathrm{cal}}={\left\{\left(x_{j},y_{j}\right)\right\}}_{j=1}^{q}.$$

A classifier (e.g., logistic regression) is then trained only on $D_{\mathrm{proper}}$. For a sample *x* and candidate label *y*, the fitted model returns a probability vector $\hat{p}\left(x\right)$, where ${\hat{p}}_{y}\left(x\right)$ denotes the probability assigned to class *y*.

Then, the calibration statistic, nonconformity score, can be computed to reflect how well a particular (*x*, *y*) combination conforms to the calibration samples. Here, we used a nonconformity score formulation that has been validated previously across multiple biomedical AI applications [16,18]:

$$\alpha \left(x,y\right)=0.5-\frac{{\hat{p}}_{y}\left(x\right)-\max_{y^{\prime }\neq y}{\hat{p}}_{y^{\prime }}\left(x\right)}{2}.$$

The conformal *p*-value for each candidate label was then computed from the empirical distribution of calibration nonconformity scores as

$$p_{y}\left(x\right)=\frac{\left|\{j:\alpha_{j}\geq \alpha \left(x,y\right)\}\right|+1}{q+1},$$

**Figure 1:**
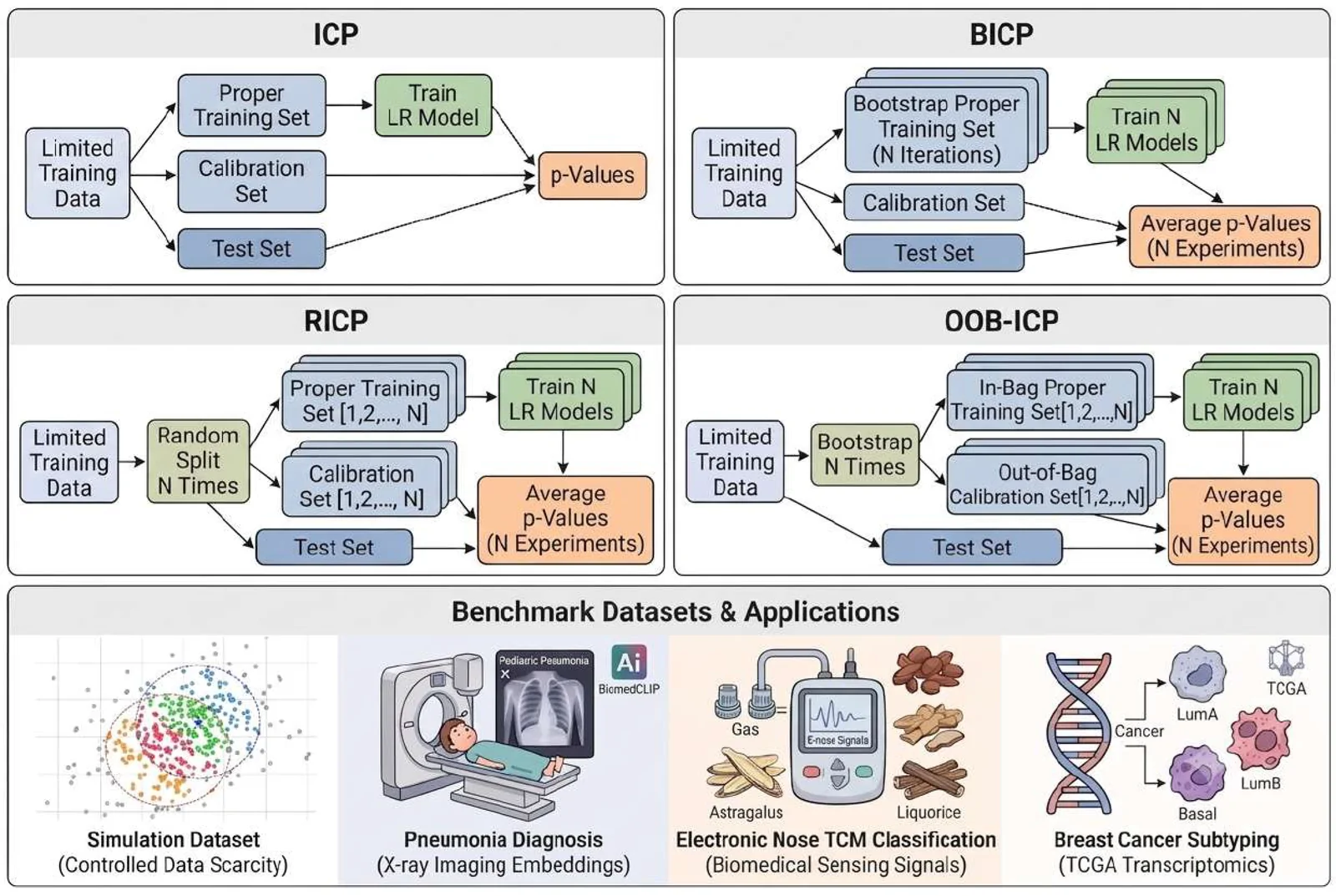
Workflow of the baseline ICP method and the three proposed robust ICP variants for prediction-reliability assessment across biomedical sensing and measurement applications. ICP uses one-time proper-training/calibration split; BICP bootstraps the proper-training set while keeping calibration fixed; RICP introduces multiple randomized proper-training/calibration partitioning, with aggregate conformity scores obtained by averaging the iteration-specific conformal *p*-values; OOB-ICP uses in-bag samples for model fitting and out-of-bag samples for calibration in each bootstrapping iteration, with aggregate conformity scores obtained by averaging the iteration-specific conformal *p*-values.

The standard ICP method uses a single proper-training/calibration split. Within each training pool, the proper-training/calibration partition was stratified-sampled by class. For the simulation and PneumoniaMNIST experiments, each sampled training set was divided into 80% proper-training and 20% calibration subsets. For alternative herbal medicine classification, the 40 available training samples per class were divided into 30 proper-training and 10 calibration samples per class. For breast cancer subtyping, each outer training partition was further divided into approximately 67% proper-training and 33% calibration subsets. In limited-data settings, standard ICP can be unstable because both the fitted model and the empirical calibration distribution depend on one-time sampled partition. Here, we proposed several ICP variants to improve the robustness of prediction reliability with statistical randomization (Figure 1):

**Bootstrapped ICP (BICP):** BICP repeatedly resamples the proper-training set with replacement while keeping the calibration set fixed [23,24]. In each bootstrap iteration, a separate classifier is trained on the resampled proper-training set, and conformal *p*-values are computed using the same calibration set. The iteration-specific conformal *p*-values are then averaged across bootstrap iterations to obtain an aggregate conformity score for each candidate label. Because arithmetic averaging of dependent conformal *p*-values does not in general preserve finite-sample *p*-value validity, this aggregated quantity is not interpreted as an exact conformal *p*-value. This design mainly aims to reduce model-fitting instability caused by small proper-training samples. In this study, we bootstrapped for 100 times.

**Randomized ICP (RICP):** RICP repeatedly repartitions the available training data into proper-training and calibration subsets. Each partition produces iteration-specific conformal *p*-values, which are averaged across randomized partitions to obtain the aggregate conformity score. This strategy targets split-induced instability under limited-data conditions; the aggregated score is used as a prediction-reliability measure rather than interpreted as an exact conformal *p*-value. In this study, we repartitioned 100 times.

**Out-of-Bag ICP (OOB-ICP):** OOB-ICP applies bootstrapping to the available training pool. In each bootstrap iteration, in-bag samples are used for classifier fitting and the corresponding out-of-bag samples for calibration. The resulting iteration-specific conformal *p*-values are averaged to obtain the aggregate conformity score for each candidate label. This procedure repeatedly reuses the limited training pool while maintaining separation between model fitting and calibration within each iteration. As with BICP and RICP, the aggregated score is not assumed to retain the exact finite-sample validity of an individual conformal *p*-value. In this study, we bootstrapped for 100 times.

**Classifier implementation, preprocessing, and reproducibility:** For the simulation, PneumoniaMNIST and alternative herbal medicine experiments, the base classifier was implemented as a scikit-learn pipeline consisting of StandardScaler followed by logistic regression with $L_{2}$ regularization, a regularization parameter of C=1.0, and a maximum of 5000 iterations. The StandardScaler parameters were estimated only from the model-fitting subset supplied to the classifier in each ICP iteration (i.e., the proper-training, bootstrap, or in-bag samples, depending on the method), and the fitted transformation was subsequently applied to the corresponding calibration subset and test samples. As a result, all data-dependent preprocessing and feature-selection steps were fitted only on the current model-fitting proper-training set and were subsequently applied to calibration and test data, preventing information leakage from the held-out samples. For the breast cancer subtyping, considering the high feature dimensionality and class imbalance, the classifier used $L_{1}$ regularization for feature selection, class-balanced weights, C=1.0, and a maximum of 1000 iterations. A base random seed of 42 was used throughout the experiments. Outer data splitting, repeated training-set sampling, proper-training/calibration repartitioning, and bootstrap resampling used deterministic repeat- and method-specific seeds derived from this base seed. The complete seed-generation procedures and implementation parameters are provided in the released reproducible code. It should be noted that no hyperparameter tuning for the classifier or data-driven model selection was performed because we aim to test the prediction reliability of different ICP variants with the base classifiers fixed.

**Ensemble-size control baselines:** To examine whether the performance gains of the proposed randomized ICP methods could be explained solely by the use of multiple fitted models and the ensembling effect, we additionally evaluated two nonconformal classification baselines on the PneumoniaMNIST dataset. PneumoniaMNIST was selected as a real-world, relatively large binary-classification testbed for this comparison. The first baseline, LR, consisted of a single logistic regression model trained directly on the entire sampled training set. The second baseline, Bootstrap-LR, served as a compute-matched bagging control: the entire sampled training set was bootstrapped 100 times, one logistic regression model was fitted to each bootstrap sample, and the resulting class probabilities were averaged across the 100 models. Specifically, for candidate class *k*,

$$\bar{P}\left(Y=k|x\right)=\frac{1}{B}\sum_{b=1}^{B}P_{b}\left(Y=k|x\right),\;B=100.$$

The class with the largest averaged probability was used as the final prediction. Both baselines used the same sampled training sets and the same fixed PneumoniaMNIST test set as the conformal prediction methods.

For each robust ICP variant, the conformal *p*-values obtained from individual resampling or repartitioning iterations were aggregated across *B* repetitions. Specifically, for candidate label *y*, we define the aggregate conformity score as

(1) $$s_{y}\left(x\right)=\frac{1}{B}\sum_{b=1}^{B}p_{y,b}\left(x\right),$$

where $p_{y,b}\left(x\right)$ denotes the conformal *p*-value obtained for candidate label *y* in the *b*-th iteration. The corresponding aggregate credibility score is then defined as

(2) $$C_{\mathrm{agg}}\left(x\right)=\max_{y}s_{y}\left(x\right)$$

For standard ICP, credibility retains its conventional interpretation as the maximum conformal *p*-value across candidate labels. For BICP, RICP, and OOB-ICP, however, the arithmetic mean of the iteration-specific conformal *p*-values is not assumed to preserve exact finite-sample conformal validity. Therefore, $s_{y}\left(x\right)$ and $C_{\mathrm{agg}}\left(x\right)$ are interpreted as an aggregate conformity score and an aggregate prediction-reliability score, respectively, rather than as an exact conformal *p*-value or a formally calibrated uncertainty measure. Their practical reliability and empirical prediction-set coverage are evaluated experimentally in the following sections.

### 2.3. Evaluation of Robust Conformal Prediction Methods

#### 2.3.1. Experiment Design with Randomness in Training Data

To systematically evaluate the performance and robustness of the proposed conformal prediction methods under randomness and limited training data, we designed a series of controlled experiments under varying data availability conditions.

For the simulation and pediatric pneumonia diagnosis datasets, which have relatively larger sample sizes, we used them as the “controlled data-scarcity testbeds”. For these two datasets, a fixed test set was firstly constructed and kept unchanged across repeated experiments, while training sets of size n=100, 200, 400, and 800 were sampled from the entire available training pool. For each level of training data scarcity, the sampling process was repeated 10 times to evaluate the robustness of different CP methods under perturbations of sampled training data. In contrast, the alternative herbal medicine classification and breast cancer subtyping datasets were treated as real-world small-sample biomedical tasks. Therefore, no additional downsampling was applied to these two datasets. Instead, to test the robustness of our method, 10 repeated stratified train–test splits were used to preserve class distributions and evaluate method robustness under naturally limited data conditions.

Under this experiment design, we evaluated each method from three complementary perspectives: overall classification performance, prediction-reliability discrimination, and credibility-score stability, which are introduced in details as follows:

#### 2.3.2. Model Classification Performance Evaluation

We first evaluated predictive performance using standard classification metrics, including accuracy and macro-average F1-score. These metrics quantify the classification model fitted on the ICP proper-training set to correctly classify samples across different datasets and class distributions.

#### 2.3.3. Prediction-Reliability Discrimination

To evaluate the ability of the credibility-based reliability score to discriminate between correct and incorrect predictions, we examined the relationship between credibility scores and prediction correctness: we first compared the estimated credibility values between correct and incorrect predictions. An informative prediction-reliability score should assign higher credibility scores to correct predictions and lower credibility to incorrect predictions. For binary-classification datasets, we further examined the credibility values of the true-positive (TP), true-negative (TN), false-positive (FP), and false-negative (FN) groups. The mean credibility for each group was computed and compared.

Secondly, to better quantify the correctness of predictions with different levels of credibility scores, we grouped test samples into five equal-frequency credibility bins and computed accuracy and F1-score within each bin. A reliable credibility score should produce a monotonically positive trend: higher credibility bins should have better predictive performance.

#### 2.3.4. Stability of Credibility-Based Reliability Scores

To assess the stability of credibility-based reliability scores, we analyzed the variability of credibility scores for individual test samples across 10 parallel experiments with random training data sampling. To be specific, for each method, each test sample is attached with multiple credibility values obtained from 10 random sampling experiments. We computed the mean and standard deviation of credibility for each sample across repetitions. A robust prediction-reliability assessment method is expected to produce low variability in credibility scores, indicating consistent reliability scores despite randomness in the training data. For the fixed-test-set simulation and pneumonia diagnosis tasks, we evaluated stability by measuring how much the final credibility assigned to the same test sample changed across repeated outer experiments. Let $c_{i,m}^{\left(r\right)}$ denote the final credibility of test sample *i* from method *m* in the repeat *r*, where r=1,…,R and R=10. For BICP, RICP, and OOB-ICP, $c_{i,m}^{\left(r\right)}$ is the final credibility after the method-specific resampling or repartitioning procedure. The across-repeat standard deviation was computed as

$${\mathrm{SD}}_{i,m}=\sqrt{\frac{1}{R}\sum_{r=1}^{R}{\left(c_{i,m}^{\left(r\right)}-{\bar{c}}_{i,m}\right)}^{2}},\;{\bar{c}}_{i,m}=\frac{1}{R}\sum_{r=1}^{R}c_{i,m}^{\left(r\right)}.$$

Lower ${\mathrm{SD}}_{i,m}$ indicates that the method is less sensitive to perturbations in the sampled training data and therefore produces more stable credibility-based reliability scores.

#### 2.3.5. Empirical Evaluation of Prediction-Set Coverage

To further characterize the empirical coverage behavior of the conformal prediction methods as the amount of labeled training data increased, we conducted an additional sequential validation experiment using PneumoniaMNIST. The released training and validation partitions were combined to form the available sample pool, while the released test partition was not used in this experiment.

The experiment started with an initial labeled training pool of n=100. At each update, the current model was used to predict the next batch of 10 previously unseen samples. The true labels of these 10 samples were withheld during prediction and were revealed only after all samples in the batch had been evaluated. The 10 newly labeled samples were then added to the training pool, after which the model was refitted for the next batch. This procedure was repeated until the labeled training pool reached n=5000, resulting in a total of 4900 sequentially evaluated samples.

The combined PneumoniaMNIST training and validation pool contained 5232 samples, including 1349 Class-0 (normal) and 3883 Class-1 (pneumonia) samples. The initial labeled pool of 100 samples was selected by stratified sampling using the fixed random seed 42 and contained 26 Class-0 and 74 Class-1 samples. The remaining samples were then randomly permuted once using a NumPy random generator with seed 1042 (42+1000), and the first 4900 samples in this fixed randomized sequence formed the sequential evaluation stream. This stream contained 1269 Class-0 and 3631 Class-1 samples and was shared by all four evaluated methods. The remaining 232 samples in the combined training and validation pool were not used in the sequential experiment.

We evaluated ICP, BICP, RICP, and OOB-ICP at significance levels ϵ=0.10 and ϵ=0.20. For standard ICP, candidate-label inclusion was determined using the conventional conformal *p*-value $p_{y}\left(x\right)$. For BICP, RICP, and OOB-ICP, candidate-label inclusion was determined using the corresponding aggregate conformity score $s_{y}\left(x\right)$. For notational convenience, we denote either quantity by $q_{y}\left(x\right)$ and define the prediction set as

$$\Gamma_{\epsilon }\left(x\right)=\left\{y:q_{y}\left(x\right)>\epsilon \right\}.$$

An error was recorded when the true label was not included in the corresponding prediction set. Let *t* denote the cumulative number of sequentially evaluated samples. The cumulative number of observed errors was compared with the nominal reference line ϵt. Accordingly, an empirical error rate close to or below ϵ corresponds to empirical coverage close to or above the nominal level 1−ϵ.

BICP, RICP, and OOB-ICP each used 20 internal resampling or repartitioning iterations per batch for computational efficiency, and the iteration-specific conformal quantities were averaged before constructing prediction sets.

For standard ICP, the ϵt reference line retains its conventional conformal significance-level interpretation under the usual assumptions. For BICP, RICP, and OOB-ICP, however, arithmetic averages of iteration-specific conformal *p*-values are treated as aggregate conformity scores and are evaluated empirically; they are not assumed to inherit the exact finite-sample validity guarantee of conventional conformal *p*-values. In addition to marginal empirical coverage, class-conditional empirical coverage and prediction-set size were evaluated to characterize their prediction-set behavior.

#### 2.3.6. Statistical Testing

To quantitatively assess differences in predictive performance and prediction-reliability assessment, we conducted 10 repeated experiments: with randomly sampled training data for the simulation dataset and PneumoniaMNIST dataset, and with randomly partitioned train/test splits for alternative herbal medicine classification and breast cancer subtyping. For classification accuracy and macro-average F1-score, paired *t*-tests with Holm correction were performed to compare each robust ICP variant with the standard ICP baseline for the first two datasets with fixed test sets, while Nadeau–Bengio corrected resampled paired *t*-tests plus Holm correction was used for the alternative herbal medicine classification and breast cancer subtyping (with 10 random partitions of test sets that may have overlapped samples across 10 repeats).

For the accuracy and macro-average F1-score analyses, the effect size for each comparison was defined as the mean paired performance difference, $\Delta =M_{\mathrm{method}}-M_{\mathrm{ICP}}$, where *M* denotes the corresponding performance metric. For each dataset and each performance metric separately, the multiplicity family consisted of the three prespecified comparisons of BICP versus ICP, RICP versus ICP, and OOB-ICP versus ICP, and Holm adjustment was applied across these three comparisons. The corresponding effect sizes, 95% confidence intervals, exact unadjusted *p*-values, and exact Holm-adjusted *p*-values are reported in the Supplementary Materials. For the simulation and PneumoniaMNIST experiments, inference was performed across repeated training-set samplings while the held-out test set remained fixed. Accordingly, the resulting inferential statistics on these two datasets are conditional on that particular held-out test set and should not be interpreted as incorporating variability arising from repeated test-set sampling.

For the correct-versus-incorrect credibility analysis, the mean credibility of correct and incorrect predictions was first calculated separately within each outer repetition, and the resulting within-repeat differences were analyzed using the Nadeau–Bengio corrected resampled paired *t*-test. This correction accounts for the dependence introduced by repeated and overlapping resampling, with the test-to-training ratio determined according to the outer evaluation design of each dataset. To account for multiple comparisons across the planned correct-versus-incorrect credibility comparisons, *p*-values were further adjusted using the Holm procedure. For the two binary classification tasks, credibility distributions were further examined descriptively within the true-positive (TP), true-negative (TN), false-positive (FP), and false-negative (FN) subgroups.

## 3. Results

### 3.1. Model Classification Performance

Table 2 summarizes the overall predictive performance in terms of classification accuracy, reported as mean ± standard deviation over repeated experiments, while results on the macro-average F1-score are shown in Table S1.

Overall, three robust conformal prediction methods (BICP, RICP and OOB-ICP) generally outperformed the standard ICP baseline. Among them, OOB-ICP showed the best overall classification performance, achieving the highest accuracy on most testing scenarios (three levels of data scarcity for X-ray-based pneumonia diagnosis and the electronic-nose-based herbal medicine classification). In contrast, RICP achieved the best classification performance on the RNA-seq molecular measurement breast cancer subtyping (mean accuracy: 0.890) task with the OOB-ICP trailing behind slightly (mean accuracy: 0.889). However, the standard ICP method consistently showed the lowest or near-lowest classification performance across datasets (except that on the simulation, all four methods reached a similar level of performance), suggesting that the use of a single proper-training/calibration split may limit predictive performance under data scarcity.

To further determine whether these improvements could be attributed solely to the use of multiple fitted models and the ensembling effect, we compared the conformal prediction methods with a single LR model and a 100-model Bootstrap-LR ensemble on PneumoniaMNIST (Table S2). Across all evaluated training-set sizes, RICP and OOB-ICP achieved higher accuracy and macro-F1 scores than both LR and Bootstrap-LR, suggesting that their performance gains cannot be explained solely by ensemble size and instead reflect additional benefits of the proposed resampling and calibration strategies. Additionally, summary confusion matrices are provided in Table S3 to characterize the distribution of error types.

**Figure 2:**
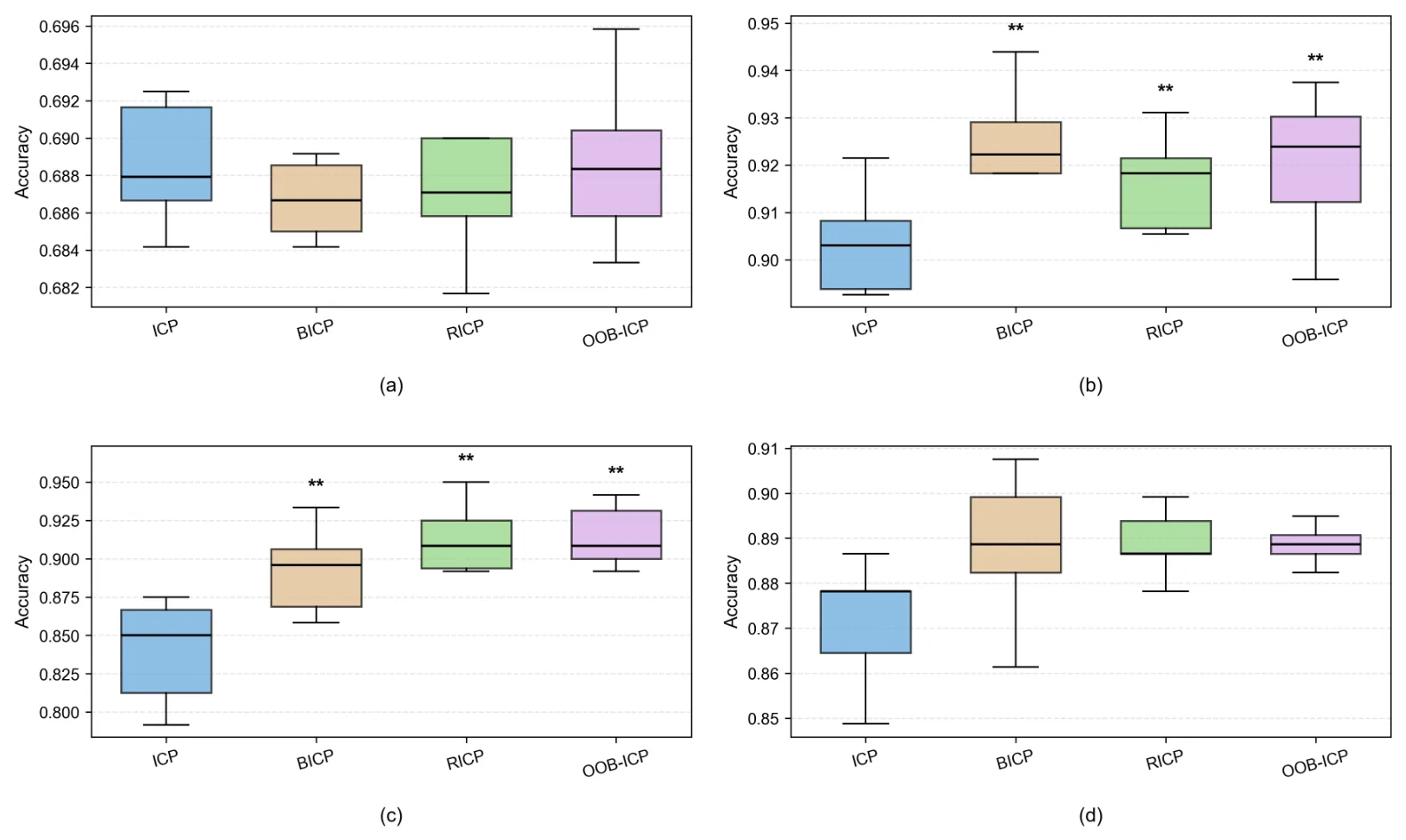
Boxplots of classification accuracy for the four conformal prediction methods across the four benchmark datasets: (**a**) simulation (*n* = 200), (**b**) pneumonia diagnosis (*n* = 200), (**c**) alternative herbal medicine classification based on electronic nose, and (**d**) breast cancer subtyping based on RNA sequencing. Statistically significant improvement against the baseline ICP is marked: *: *p* < 0.05, **: *p* < 0.01, ***: *p* < 0.001, paired *t*-tests with Holm correction for the first two datasets with fixed test sets across 10 repeats, Nadeau–Bengio corrected resampled paired *t*-test with Holm correction for the last two datasets with randomly split test sets across 10 repeats.

**Figure 3:**
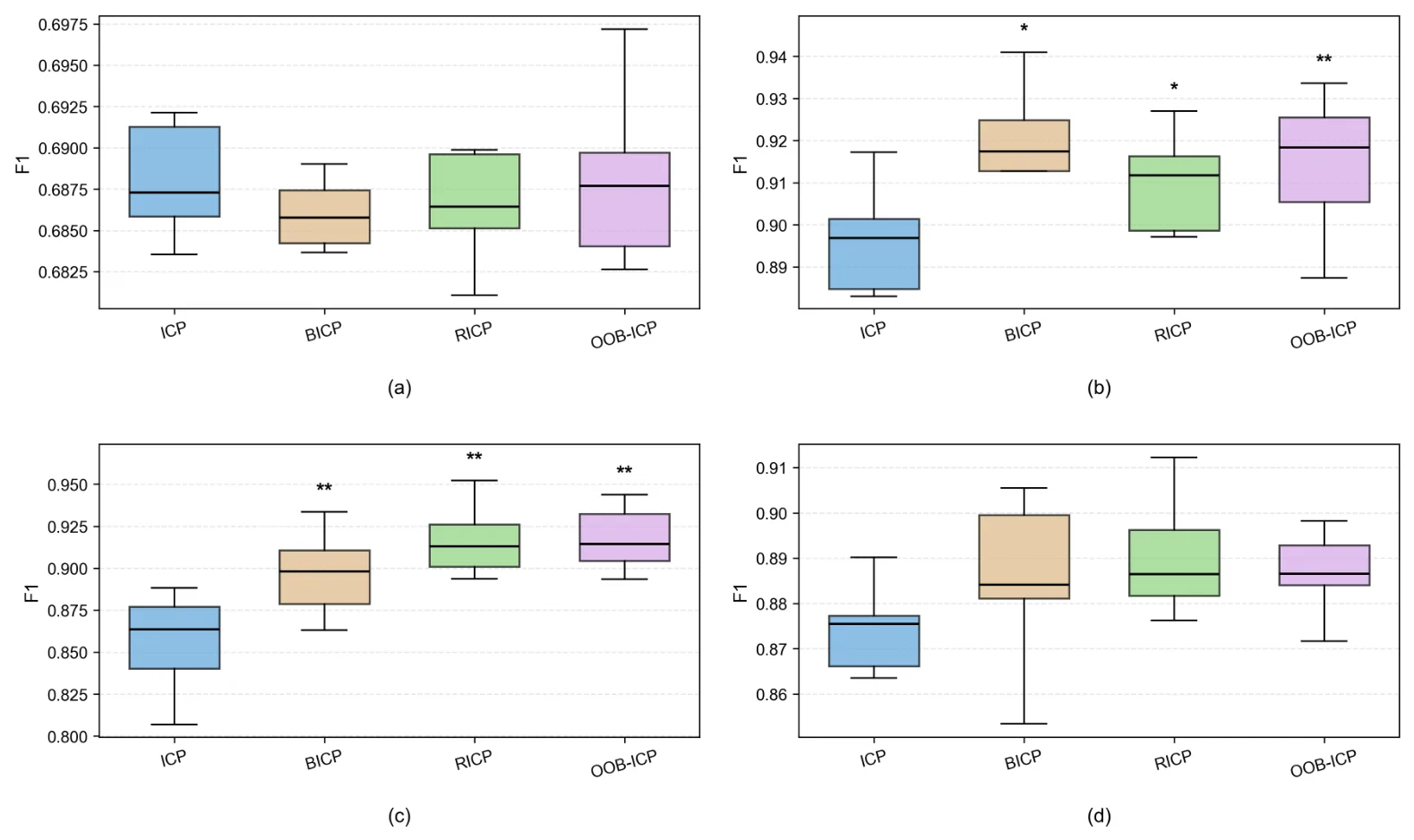
Boxplots of F1-score for the four conformal prediction methods across the four benchmark datasets: (**a**) simulation (*n* = 200), (**b**) pneumonia diagnosis (*n* = 200), (**c**) alternative herbal medicine classification based on electronic nose, and (**d**) breast cancer subtyping based on RNA sequencing. Statistically significant improvement against the baseline ICP is marked: *: *p* < 0.05, **: *p* < 0.01, ***: *p* < 0.001, paired *t*-tests with Holm correction for the first two datasets with fixed test sets across 10 repeats, Nadeau–Bengio corrected resampled paired *t*-test with Holm correction for the last two datasets with randomly split test sets across 10 repeats.

The boxplots of classification accuracy shown in Figure 2 further support the observations in Table 2. For the Simulation (Figure 2a), with a relatively easier task, four methods reached a similar accuracy level, with no statistically significant difference. On pneumonia diagnosis and alternative herbal medicine classification (Figure 2b,c), all three robust ICP methods were significantly better than the standard ICP (p<0.01), while no statistical significance was observed on the breast cancer subtyping. Among the three robust ICP methods, OOB-ICP reached an overall highest accuracy. A similar overall trend was observed in terms of F1-score, as shown in Figure 3.

The complete statistical results for the accuracy and macro-average F1-score comparisons, including mean paired performance differences, 95% confidence intervals, exact unadjusted *p*-values, and exact Holm-adjusted *p*-values, are provided in Table S4.

Taken together, these results demonstrate that robust conformal prediction methods achieved comparable or better downstream classification performance under limited-data settings, with OOB-ICP showing the strongest and most consistent overall advantage. Although repeated model fitting and aggregation may contribute to these gains, the compute-matched Bootstrap-LR comparison indicates that ensemble size alone does not fully explain the improvements observed for RICP and OOB-ICP. The primary focus of the proposed methods, however, remains the robustness of prediction reliability, which is evaluated in the following sections.

### 3.2. Prediction-Reliability Discrimination

We next evaluated whether the credibility-based reliability scores were informative of prediction correctness. Correctly predicted samples generally received higher credibility scores than incorrectly predicted samples, indicating that the scores provided meaningful discrimination between predictions with different levels of empirical reliability.

**Figure 4:**
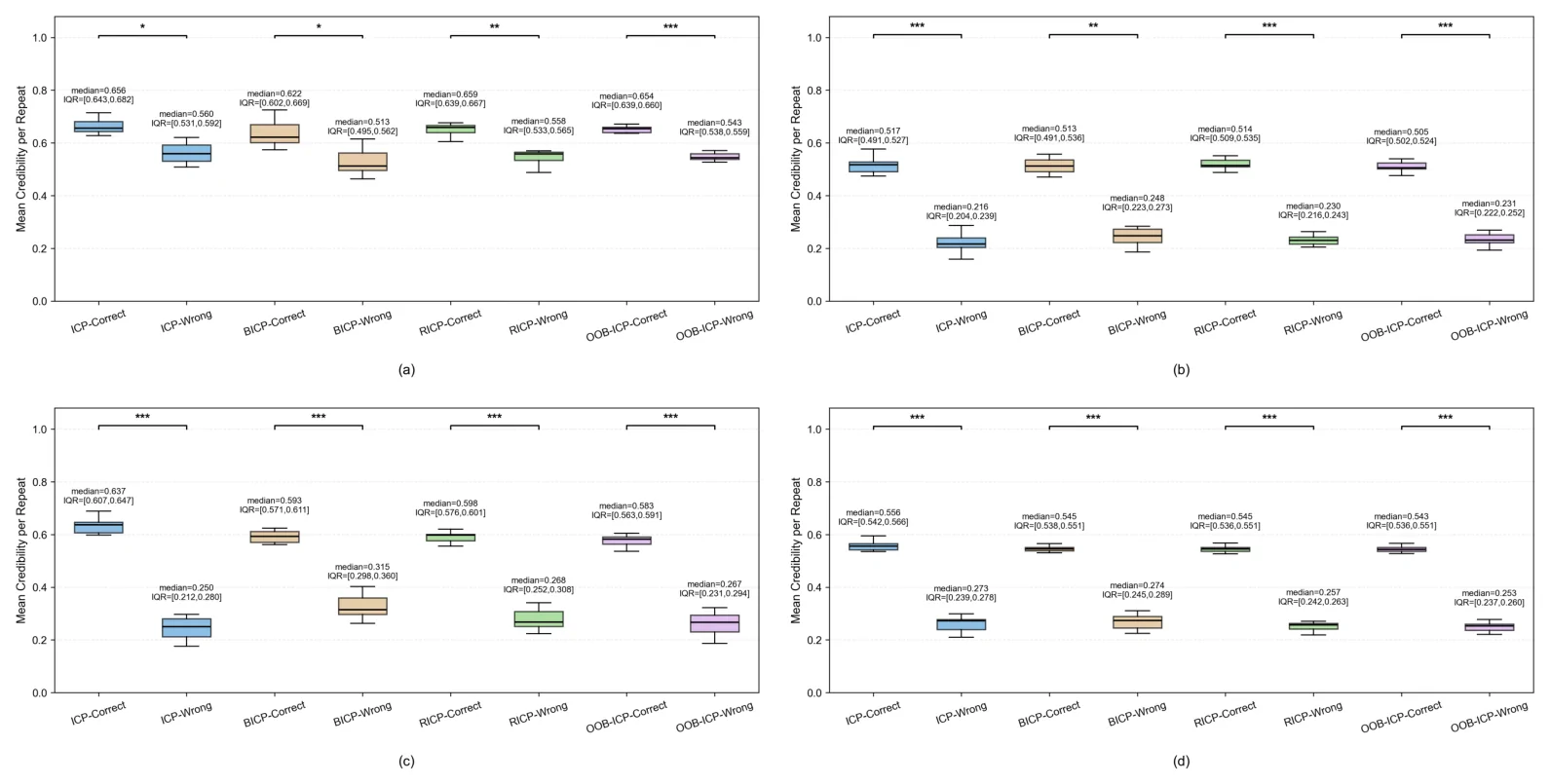
Mean conformal credibility scores for correct and incorrect predictions across 10 repeated experiments. The four subplots correspond to (**a**) Simulation (n=200), (**b**) pneumonia diagnosis (n=200), (**c**) alternative herbal medicine classification based on electronic nose, and (**d**) breast cancer subtyping based on RNA sequencing. Higher conformal credibility score in correctly predicted groups than in incorrectly predicted groups indicates better prediction-reliability discrimination. Statistical significance was assessed using the Nadeau–Bengio corrected resampled paired *t*-test with Holm correction for multiple comparisons: * $p_{\mathrm{adj}}<0.05$, ** $p_{\mathrm{adj}}<0.01$, and *** $p_{\mathrm{adj}}<0.001$. The full results, including effect sizes and statistical significance, are provided in Table S5.

As shown in Figure 4, credibility exhibited clear differences across prediction outcome groups: correctly predicted samples were assigned higher conformal credibility scores than incorrectly predicted samples, indicating that the estimated prediction reliability was effectively related to predictive correctness. This trend was consistently observed across the studied datasets. Notably, all comparisons shown in Figure 4 remained statistically significant after Holm correction for multiple comparisons (adjusted p<0.05) with the statistical testing details shown in Table S5. This result demonstrates that the conformal credibility score differences between correct and incorrect prediction groups were consistently statistically significant, further supporting that the credibility-based scores provide meaningful information regarding prediction correctness. The comparative benefit of the randomized methods is therefore interpreted primarily in terms of improved repeated-sampling stability rather than uniformly stronger prediction-reliability discrimination.

**Figure 5:**
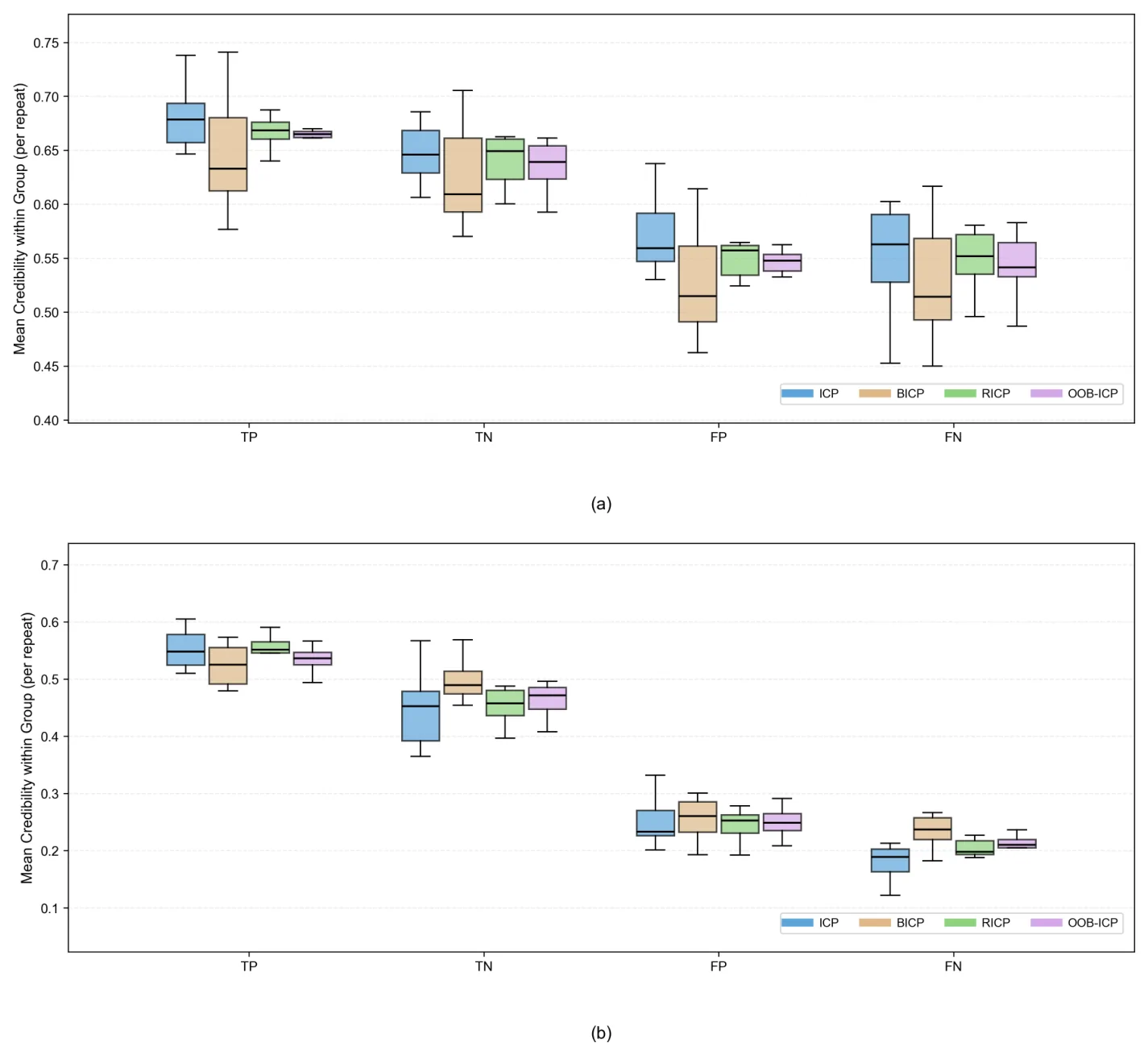
Fine-grained analysis of prediction-reliability discrimination based on TP, TN, FP, and FN groups for selected datasets. (**a**) simulation (*n* = 200), (**b**) pneumonia diagnosis (*n* = 200).

To further examine this relationship for the binary classification tasks, we visualized the TP, TN, FP, and FN groups separately for selected datasets and methods in Figure 5. For the simulation (Figure 5a), all four methods showed a clear pattern in which TP and TN groups had higher conformal credibility scores than FP and FN groups. This indicates that the credibility-based reliability scores were directionally informative: predictions that were correct (TP, TN) tended to receive stronger credibility support, whereas incorrect predictions (FN, FP) were assigned lower credibility. For the pneumonia diagnosis (Figure 5b), this separation was even more pronounced. In particular, OOB-ICP showed a very strong contrast between the correctly predicted groups and the incorrectly predicted groups, indicating that its credibility scores were more closely aligned with actual prediction quality.

Across all datasets, the evaluated methods showed clear separation between correct and incorrect predictions, although the magnitude of this separation was not consistently greater for the randomized methods than for standard ICP.

**Figure 6:**
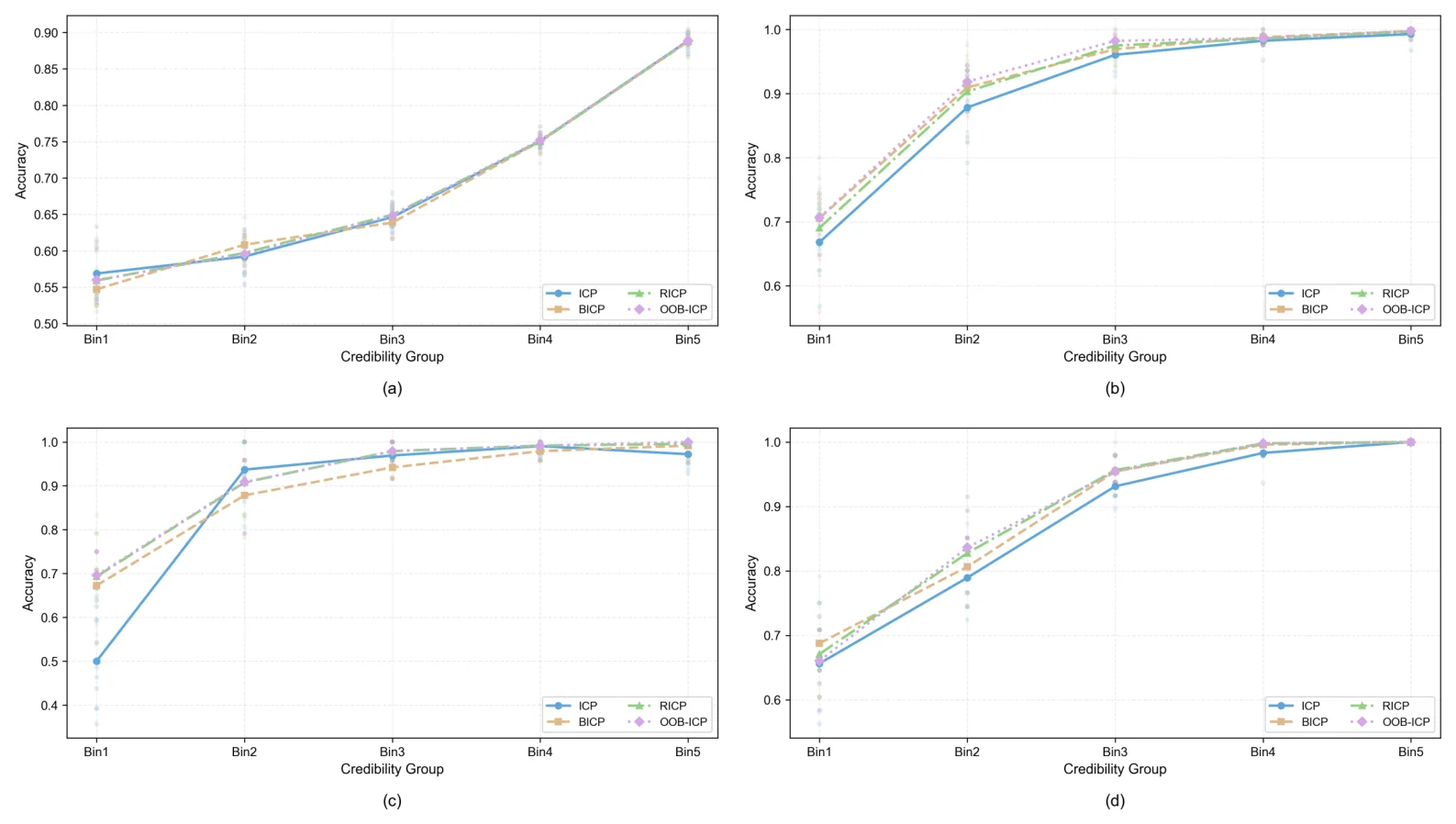
Relationship between conformal credibility score ranges and classification performance across datasets. Samples were divided into five equal-frequency bins according to credibility values, and predictive accuracy was computed within each bin. Solid markers and lines show the mean value across 10 parallel experiments. (**a**) simulation (*n* = 200), (**b**) pneumonia diagnosis (*n* = 200), (**c**) alternative herbal medicine classification based on electronic nose, and (**d**) breast cancer subtyping based on RNA sequencing.

In addition, we further evaluated prediction-reliability discrimination by grouping samples into five equal-size credibility bins and examining classification performance within each bin. As shown in Figure 6, a monotonically positive relationship was observed between conformal credibility scores and predictive performance across all datasets. In general, samples with higher conformal credibility scores were associated with higher classification accuracy, indicating that credibility provides a meaningful ranking of prediction reliability. A similar trend can be seen on the macro-average F1-score in Figure S1.

For the Simulation, accuracy increased steadily from Bin 1 to Bin 5 across all methods. The same monotonic trend was observed in the pneumonia diagnosis dataset and breast cancer subtyping, where the high-credibility bins approached near-perfect classification accuracy, particularly for RICP and OOB-ICP. However, for the electronic-nose-based herbal medicine classification, the standard ICP showed a regression in the accuracy from bin 4 to bin 5, which indicates a flaw in prediction reliability, while all robust ICP methods showed monotonically increasing accuracy from bin 1 to bin 5.

Overall, these analyses demonstrate that the credibility-based scores provide meaningful prediction-reliability information across the evaluated methods.

### 3.3. Stability of Credibility-Based Reliability Scores

**Figure 7:**
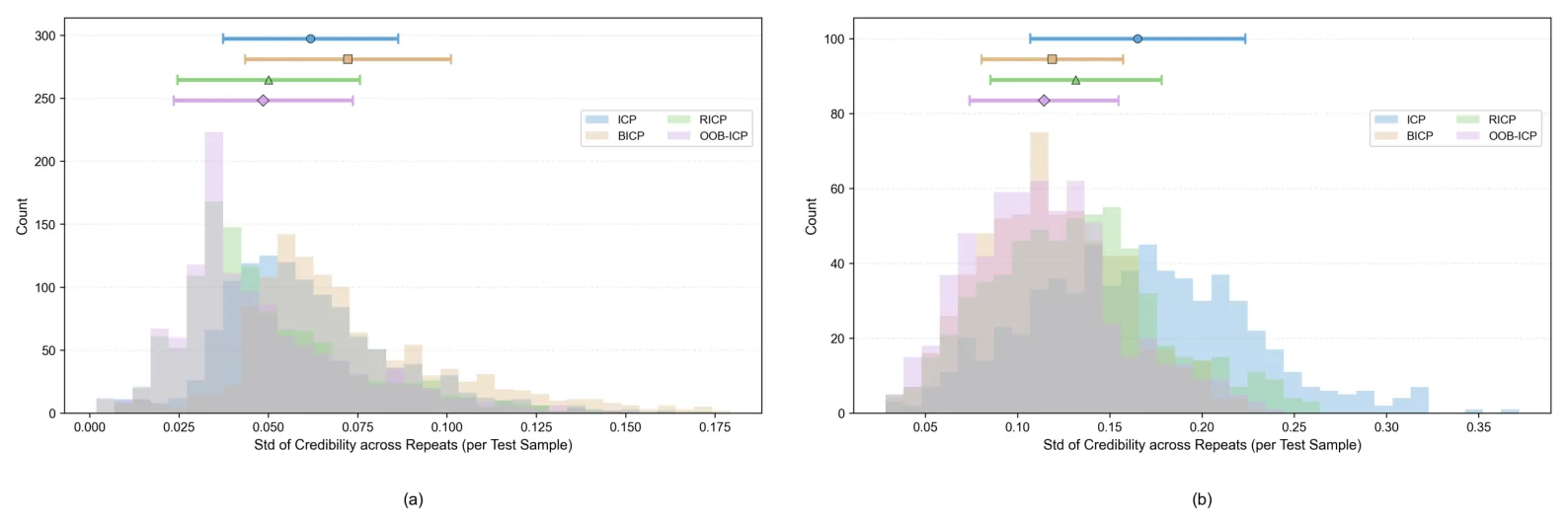
Distribution of per-sample conformal credibility score standard deviation across 10 parallel re-sampling experiments for selected datasets: (**a**) simulation (*n* = 200); (**b**) pneumonia diagnosis (*n* = 200). Narrower and more concentrated distributions indicate more stable prediction reliability under training data sampling.

Finally, we assessed the stability of credibility-based reliability by analyzing the variability of conformal credibility scores across repeated experiments. For each test sample, the standard deviation of credibility across 10 repeated runs was computed, and the distributions of these per-sample credibility standard deviations are shown in Figure 7.

For the Simulation dataset (Figure 7a) and pneumonia diagnosis (Figure 7b), OOB-ICP showed the narrowest and most concentrated distribution (purple), with most samples having very small credibility standard deviations across 10 random training sampling experiments. RICP (green) also showed a relatively concentrated low-variability distribution. By contrast, ICP (blue) in general exhibited broader distribution, indicating stronger sensitivity to the randomness induced by the single training/calibration split. BICP reduced the variability for the pneumonia diagnosis task, but its credibility scores remained less stable than those of RICP and OOB-ICP.

These findings indicate that OOB-ICP and RICP produced the most stable aggregate credibility scores under repeated training-data sampling. Together with the classification-performance results and the prediction-reliability discrimination described in earlier sections, these results support the effectiveness of robust conformal prediction strategies for biomedical AI applications under limited-data settings, with OOB-ICP showing the strongest overall performance.

### 3.4. Empirical Evaluation of Prediction-Set Coverage

**Figure 8:**
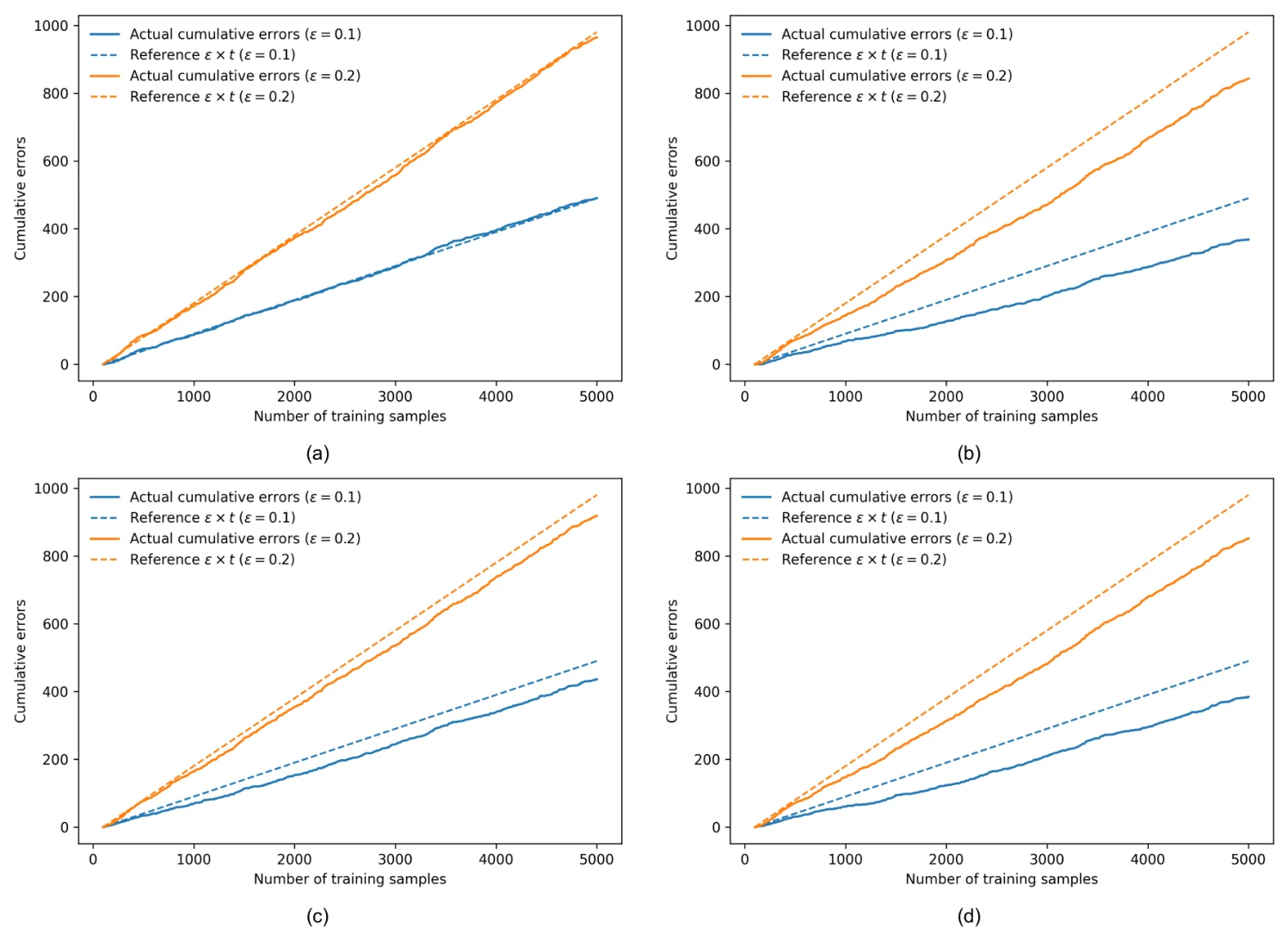
Empirical prediction-set coverage of the proposed robust ICP methods on PneumoniaMNIST. The validation started with n=100 labeled training samples and progressively increased the training pool to n=5000 by sequentially evaluating batches of 10 previously unseen samples. Solid lines show the actual cumulative prediction-set errors at ϵ=0.10 and ϵ=0.20, while dashed lines show the corresponding nominal reference values ϵt, where *t* denotes the cumulative number of sequentially evaluated samples. The four panels correspond to (**a**) ICP, (**b**) BICP, (**c**) RICP, and (**d**) OOB-ICP.

To further evaluate the empirical prediction-set coverage of the conformal prediction methods, we conducted a sequential validation experiment on PneumoniaMNIST. Starting from n=100 labeled training samples, the training pool was progressively increased to n=5000, while 4900 previously unseen samples were sequentially evaluated. Figure 8 shows the cumulative number of prediction-set errors for ICP, BICP, RICP, and OOB-ICP at ϵ=0.10 and ϵ=0.20, together with the corresponding nominal reference lines ϵt.

As summarized in Table 3, all eight method-significance-level combinations ended with empirical error rates no greater than their corresponding reference levels. Standard ICP produced 490 errors at ϵ=0.10 and 964 at ϵ=0.20, corresponding to marginal empirical coverage of 0.900 and 0.803. BICP produced 368 and 843 errors, RICP produced 436 and 918, and OOB-ICP produced 384 and 851, respectively. Thus, in this experiment BICP showed the lowest final empirical error rates (0.075 and 0.172), closely followed by OOB-ICP (0.078 and 0.174). These observations characterize empirical behavior only and do not establish a finite-sample validity guarantee for the aggregated methods.

The class-conditional results were less uniform. At ϵ=0.10, Class-0 coverage was 0.842, 0.890, 0.854, and 0.872 for ICP, BICP, RICP, and OOB-ICP, respectively, whereas Class-1 coverage was 0.920, 0.937, 0.931, and 0.939. At ϵ=0.20, the corresponding Class-0 coverage values were 0.679, 0.737, 0.685, and 0.716, while Class-1 coverage values were 0.847, 0.860, 0.857, and 0.865. Therefore, marginal empirical coverage at or above the nominal level did not imply uniform class-conditional coverage; Class 0 remained below the nominal coverage level for all four methods at both significance levels. This pattern is consistent with the pooled calibration strategy used in the experiment and should not be interpreted as establishing a class-conditional validity guarantee.

Prediction sets were predominantly singleton or empty rather than enlarged multi-label sets. At ϵ=0.10, average set sizes were 0.918, 0.950, 0.927, and 0.939 for ICP, BICP, RICP, and OOB-ICP, with singleton proportions of 0.914, 0.938, 0.926, and 0.937. At ϵ=0.20, average set sizes were 0.809, 0.834, 0.817, and 0.832, with singleton proportions of 0.809, 0.833, 0.817, and 0.832, respectively. Multiple-label prediction sets were rare in all conditions.

## 4. Discussion

In this study, we proposed three randomized ICP variants and systematically investigated whether statistical resampling can improve the informativeness and repeated-sampling stability of conformal-derived prediction-reliability scores under limited biomedical training data. Across four benchmark classification tasks spanning simulation, medical imaging, biomedical gas sensing, and RNA sequencing, credibility-based scores from all evaluated methods were informative of prediction correctness, while the proposed randomized methods generally showed reduced sensitivity to perturbations in the sampled training data. Among the evaluated approaches, OOB-ICP showed the strongest overall combination of predictive performance, reliability discrimination, and score stability.

Importantly, these findings should not be interpreted as demonstrating more accurate uncertainty quantification in a general or metrological sense. The primary quantities investigated in BICP, RICP, and OOB-ICP are aggregate conformity and credibility scores derived from repeated conformal prediction procedures. Accordingly, the principal contribution of the proposed randomization strategies is the improved repeated-sampling stability of conformal-derived prediction-reliability assessment, rather than a general improvement in uncertainty quantification or a new guarantee of conformal validity.

The present findings are particularly relevant to sensing-driven biomedical AI because reliability assessment occurs downstream of the measurement process. Biomedical sensing modalities can differ substantially in signal dimensionality, acquisition mechanism, measurement variability, and the availability of labeled samples. In the present study, these characteristics are represented by radiographic imaging, a 16-channel semiconductor electronic-nose system, and high-dimensional RNA-seq molecular measurements. The electronic-nose application provides the most direct sensor-system example: multiple semiconductor channels respond to different volatile organic compounds, while only 50 labeled samples are available for each of 12 classes. Under such limited sensor-derived data, allocating observations between model fitting and conformal calibration can materially affect the learned classifier and its resulting reliability scores. The proposed resampling strategies directly target this finite-sample and partition-induced component of variability by repeatedly reusing or repartitioning the available labeled measurements.

Importantly, the proposed methods operate at the prediction-reliability layer and therefore do not require modification of the underlying sensing hardware or acquisition process. This makes the framework potentially applicable across heterogeneous sensing pipelines once sensor or measurement signals have been transformed into features suitable for classification. At the same time, the framework does not explicitly model sensor noise, drift, device-to-device variability, or measurement uncertainty; these remain distinct sources of uncertainty that warrant dedicated investigation in future sensor-system studies.

As for rationale, standard ICP relies on one fixed proper-training/calibration split. When the available labeled training data are limited, randomness in the sampled training subset can change both the fitted classifier (from proper-training subset) and the empirical calibration distribution (calibration subset), which in turn changes the credibility assigned to the same test sample. The across-experiment variability analyzed in this study therefore reflects how sensitive a method’s final credibility output is to different realizations of limited training data. From this perspective, the weakness of standard ICP is not merely that it uses fewer samples, but that its prediction-reliability assessment can be overly dependent on a single partition of those samples.

The superior performance of OOB-ICP is likely related to its more efficient and more balanced use of available data. In OOB-ICP, each bootstrap iteration uses in-bag samples for model training and out-of-bag samples for calibration. This allows the full training pool to contribute repeatedly to both stages of the conformal prediction framework without violating the separation between model fitting and calibration. This repeated reuse of data appears to improve both predictive performance and the stability of reliability assessment. In our experiments, OOB-ICP showed the overall best classification performance, and produced the smallest across-experiment credibility variation. These findings suggest that OOB-based calibration offers an effective compromise between data efficiency and statistical reliability in small-data biomedical tasks.

Another contribution of this work is the evaluation of prediction reliability from multiple complementary perspectives. Rather than focusing only on classification accuracy, we assessed (1) whether credibility scores were aligned with actual prediction correctness, (2) whether higher credibility corresponded to better predictive performance across credibility bins, and (3) whether credibility-based reliability scores remained stable across repeated experiments with random training samples. This broader evaluation strategy is particularly important in biomedical AI, where prediction-reliability methods should not merely accompany a prediction but should provide actionable information about prediction reliability.

From an application perspective, these findings are relevant to a wide range of biomedical sensing and measurement systems coupled with machine learning analysis. In many clinical and translational sensing applications, model development is constrained by limited labeled datasets, costly acquisition or expert annotation, and heterogeneity across patients, devices, measurement sessions, or biological samples. Under such conditions, improving prediction-reliability assessment is not only a technical issue but also a matter of safety and trustworthiness. A model that can better distinguish reliable from unreliable predictions, while remaining stable under modest fluctuations in the available training data, may be more useful in assisting clinicians, prioritizing human review, and reducing the risk of overconfident incorrect predictions. The present results therefore support the potential of robust conformal prediction, particularly OOB-ICP, which provides methodological evidence that warrants evaluation in externally validated and prospective clinical settings. However, no external, prospective, or clinical-deployment validation was performed; therefore, the present study does not establish clinical safety or clinical utility.

An important distinction concerns the interpretation and validity of the quantities produced by the proposed resampling-based methods. While each individual ICP iteration produces conformal *p*-values under the corresponding assumptions, BICP, RICP, and OOB-ICP aggregate these quantities across repeated resampling or repartitioning. Arithmetic averaging of such dependent *p*-values does not, in general, preserve the exact finite-sample validity of a conventional conformal *p*-value. We therefore interpret the resulting quantities as aggregate conformity scores, and their maximum across candidate labels as an aggregate credibility score reflecting prediction reliability, rather than as exact conformal *p*-values. Consistent with this distinction, the present study evaluates their prediction-set behavior empirically by examining overall empirical coverage and class-conditional coverage across nominal error levels together with prediction-set size. Overall, the empirical coverage analysis showed favorable marginal prediction-set coverage behavior for ICP, BICP, RICP, and OOB-ICP on PneumoniaMNIST, although class-conditional coverage differed substantially between the two classes. However, the interpretation differs between standard ICP and the proposed aggregated methods. For standard ICP, the ϵt reference has the conventional conformal significance-level interpretation under the usual assumptions. For BICP, RICP, and OOB-ICP, which aggregate iteration-specific conformal quantities across repeated resampling or repartitioning procedures, the observed coverage should be interpreted as empirical prediction-set behavior and does not establish that the averaged scores inherit the exact finite-sample validity of standard conformal *p*-values. Thus, the primary contribution of the proposed randomization strategies should be understood as improving the repeated-sampling stability of conformal-derived reliability scores under data scarcity, while formal validity-preserving aggregation remains an important direction for future methodological development.

It is also important to clarify the sources of uncertainty and variability addressed in this study. The proposed robust ICP methods are not intended to provide a comprehensive decomposition of all uncertainty sources in a biomedical machine learning pipeline. Rather, they primarily target the variability of prediction-reliability scores arising from limited training data, model fitting, and the random partitioning of available samples into proper-training and calibration sets. In particular, repeated resampling and repartitioning in BICP, RICP, and OOB-ICP are designed to reduce the sensitivity of the resulting credibility-based reliability scores to specific realizations of limited training data and calibration subsets. Other sources of uncertainty, including measurement or input uncertainty arising from biomedical sensors or assays, label uncertainty due to annotation errors or inter-observer variability, distributional uncertainty caused by differences between training and deployment populations, and the explicit decomposition of predictive uncertainty into aleatoric and epistemic components, are not separately quantified in the present framework. Although these sources may indirectly influence model predictions and the resulting conformity scores, their individual contributions cannot be disentangled from the current analysis. Therefore, the improved stability observed in this study should be interpreted specifically as robustness to finite-sample, model-fitting, and calibration-partition variability, rather than as a reduction in total predictive or measurement uncertainty. Future work could extend the framework by explicitly modeling these additional uncertainty sources and investigating how they interact with conformal calibration and resampling-based reliability assessment. Future work should therefore investigate the interaction between resampling-based prediction-reliability assessment and sensor-specific uncertainty sources, including measurement noise, sensor drift, device-to-device variability, acquisition-condition changes, and missing or corrupted sensing channels. Evaluating these factors using prospectively collected multisensor datasets would provide a natural extension of the present framework toward deployment-oriented biomedical sensing systems.

This study also has several limitations that warrant further investigation. First, we used logistic regression as the base classifier throughout the experiments as a controlled methodological benchmark. Although this choice improves interpretability and comparability across datasets [25], the relative performance of the proposed methods may differ when more complex base learners, such as random forests, gradient boosting models, or deep neural networks, are used. Second, although the four benchmark tasks cover multiple biomedical data modalities, they cannot represent the full diversity of real-world biomedical AI applications. Additional validation on larger external datasets, more severe label-noise settings, and broader class imbalance scenarios would further strengthen the generalizability of the findings.

The credibility-based analyses in this study primarily assess the discriminative or ranking ability of prediction-reliability scores, rather than their probabilistic calibration. Specifically, the separation of credibility between correct and incorrect predictions and the relationship between credibility bins and predictive accuracy evaluate whether the proposed scores appropriately rank predictions according to their empirical reliability. Although the additional empirical prediction-set coverage analysis provides complementary information regarding coverage behavior, a comprehensive evaluation of selective-prediction performance would further benefit from metrics such as risk-coverage curves and error-detection AUROC/AUPRC. These analyses are beyond the scope of the present study and represent an important direction for future evaluation.

Additionally, although improved prediction-reliability assessment may ultimately be useful in biomedical decision-support settings, the present study evaluates the proposed methods retrospectively on benchmark datasets and does not include external, prospective, or clinical-deployment validation. Therefore, the present findings should not be interpreted as demonstrating clinical safety or clinical utility. Future studies involving independent external cohorts and prospective evaluation are needed to determine whether the observed improvements in reliability assessment translate into meaningful benefits in real-world clinical settings.

Furthermore, the current study focuses primarily on credibility as the core prediction-reliability metric. Future work may benefit from jointly examining confidence, prediction sets, and abstention strategies to better characterize the clinical utility of conformal prediction reliability. Finally, while the robust ICP variants reduce credibility-score variability and improve the alignment between reliability scores and predictive correctness, a formal analysis of their theoretical validity and finite-sample behavior under repeated resampling remains an important direction for future methodological work.

One additional limitation is that the present study focuses on conformal classification problems. Although the resampling principles underlying BICP, RICP, and OOB-ICP are not inherently restricted to classification, the present formulations and experiments use classification-specific nonconformity scores and credibility measures. Conceptually, analogous randomization strategies could be incorporated into conformal regression by repeatedly resampling or repartitioning the available training data, fitting a regression model within each iteration, and computing calibration residuals or other regression-specific nonconformity scores to construct prediction intervals. The aggregation of information across these repeated conformal regression procedures would, however, require careful treatment to preserve or empirically characterize coverage, analogous to the validity considerations discussed for the classification setting. Extension and systematic validation of these strategies for regression therefore remain important directions for future work.

## 5. Conclusions

This study demonstrates that statistical randomization can improve the robustness of credibility-based prediction-reliability assessment under data scarcity, a common challenge in biomedical sensing and measurement applications. Across complementary benchmarks spanning X-ray imaging, multichannel semiconductor electronic-nose sensing, RNA-seq molecular measurements, and controlled simulation, the proposed methods produced aggregate credibility scores that remained informative of prediction correctness while showing reduced sensitivity to training-data perturbations compared with scores obtained from a single ICP partition. Importantly, these aggregate scores are interpreted as conformal-derived reliability measures rather than exact conformal *p*-values, and their prediction-set coverage is therefore characterized empirically. Among the evaluated strategies, OOB-ICP showed a strong overall combination of predictive performance, reliability discrimination, and repeated-sampling stability. These results support statistical randomization as a practical approach for stabilizing conformal-derived prediction-reliability assessment in sensing-driven biomedical AI when labeled measurements are limited.

## Figures and Tables

**Table 1:** Summary of the four benchmark datasets used in this study.

Task	Sensing Modality	Sample Size	Feature Representation	Classes
Simulation	Controlled synthetic benchmark	6000	2-dimensional synthetic Gaussian features	2
Pediatric pneumonia diagnosis	X-ray radiographic imaging	5856	BiomedCLIP image embeddings from 28×28 Chest X-ray images	2 [1]
Alternative herbal medicine classification	16-channel semiconductor gas-sensor array	600	128-dimensional electronic-nose signal features	12 [4]
Breast cancer subtyping	RNA-seq molecular measurement	1096 original cohort; 949 analyzed	31,098 RNA-seq gene-expression features	5 original subtypes; 3 analyzed (LumA, LumB, Basal) [7]

**Table 2:** Classification accuracy of the four conformal prediction methods across the four benchmarking datasets. Results are reported as mean ± standard deviation over repeated experiments. The best-performing method for each dataset and training set size is shown in bold.

Dataset	ICP	BICP	RICP	OOB-ICP
Simulation (n=100)	0.685±0.004	0.689±0.006	0.689±0.004	0.688±0.005
Simulation (n=200)	0.689±0.005	0.686±0.005	0.689±0.005	0.689±0.005
Simulation (n=400)	0.690±0.008	0.691±0.005	0.691±0.006	0.691±0.006
Simulation (n=800)	0.692±0.004	0.693±0.005	0.693±0.005	0.693±0.004
Pneumonia diagnosis (n=100)	0.899±0.020	0.898±0.026	0.906±0.017	0.910±0.015
Pneumonia diagnosis (n=200)	0.896±0.021	0.914±0.027	0.910±0.020	0.918±0.018
Pneumonia diagnosis (n=400)	0.902±0.028	0.916±0.019	0.911±0.021	0.917±0.018
Pneumonia diagnosis (n=800)	0.906±0.016	0.917±0.010	0.910±0.012	0.916±0.011
Alternative herbal medicine classification	0.842±0.030	0.893±0.024	0.913±0.020	0.915±0.018
Breast cancer subtyping	0.872±0.012	0.889±0.013	0.890±0.009	0.889±0.009

**Table 3:** Empirical coverage of ICP, BICP, RICP, and OOB-ICP in the sequential PneumoniaMNIST validation experiment. A prediction-set error was recorded when the true label was not included in the prediction set at significance level ϵ.

Method	ϵ	Errors	Error Rate	Empirical Coverage	Nominal Coverage
ICP	0.10	490	10.00%	90.00%	90%
ICP	0.20	964	19.67%	80.33%	80%
BICP	0.10	368	7.51%	92.49%	90%
BICP	0.20	843	17.20%	82.80%	80%
RICP	0.10	436	8.90%	91.10%	90%
RICP	0.20	918	18.73%	81.27%	80%
OOB-ICP	0.10	384	7.84%	92.16%	90%
OOB-ICP	0.20	851	17.37%	82.63%	80%

## Data Availability

The datasets used in this study can be found in their respective references in Materials and Methods. The code to reproduce the experiments and figures is available at https://github.com/ZhouYuqi11/Robust-ICP-Biomedical-AI (accessed on 1 October 2026).

## Supplementary Materials

The following supporting information can be downloaded at https://www.mdpi.com/article/10.3390/s26206513/s1, Table S1. F1-score of the four conformal prediction methods across the four benchmark datasets. Table S2. Comparison of the four conformal prediction methods with single-model and computematched ensemble baselines on PneumoniaMNIST. Table S3. Mean confusion-matrix entries for PneumoniaMNIST at *n* = 200 across 10 repeated experiments. Table S4. Effect sizes, 95% confidence intervals, and exact *p*-values for the classification accuracy and macro-averaged F1-score comparisons with ICP. Table S5. Effect sizes and corrected 95% confidence intervals for the correct-versus-incorrect credibility comparisons. Figure S1. F1-score across five equal-frequency credibility groups.
