# Supplementary material for: Robust Prediction-Reliability Assessment in Biomedical Sensing and Classification Under Data Scarcity Using Conformal Prediction and Statistical Randomization
Source: Sensors (Basel). 2026 Oct 14;26(20):6513. doi: 10.3390/s26206513 (PMC13709015; doi:10.3390/s26206513)
Supplement: Supplementary file 1 [file sensors-26-06513-s001.zip › sensors-4471183-supplementary.pdf]

Article

Robust Prediction-Reliability Assessment in Biomedical Sensing and Classification Under Data Scarcity Using Conformal Prediction and Statistical Randomization

Yuqi Zhou [1] and Xianghao Zhan [2,*]

[1] Department of Statistics, Zhejiang University, Hangzhou, China 310000, zhouyuqi@zju.edu.cn
[2] Department of Biomedical Data Science, Stanford University, Stanford, CA 94305, USA
* Correspondence: xzhan96@stanford.edu

Supplementary Results

Table S1. F1-score of the four conformal prediction methods across the four benchmark datasets. Results are reported as mean ± standard deviation over repeated experiments. The best-performing method for each dataset and sample size is shown in bold.

Dataset	ICP	BICP	RICP	OOB-ICP
Simulation ($n = 100$)	0.685 ± 0.004	$\mathbf{0.689 \pm 0.006}$	0.688 ± 0.005	0.688 ± 0.005
Simulation ($n = 200$)	$\mathbf{0.689 \pm 0.005}$	0.686 ± 0.005	0.688 ± 0.005	0.688 ± 0.005
Simulation ($n = 400$)	0.690 ± 0.008	0.690 ± 0.005	0.691 ± 0.007	$\mathbf{0.691 \pm 0.006}$
Simulation ($n = 800$)	0.692 ± 0.004	$\mathbf{0.693 \pm 0.005}$	0.693 ± 0.005	0.693 ± 0.004
Pneumonia diagnosis ($n = 100$)	0.894 ± 0.020	0.894 ± 0.026	0.901 ± 0.018	$\mathbf{0.905 \pm 0.016}$
Pneumonia diagnosis ($n = 200$)	0.888 ± 0.024	0.907 ± 0.031	0.903 ± 0.023	$\mathbf{0.912 \pm 0.020}$
Pneumonia diagnosis ($n = 400$)	0.892 ± 0.033	0.909 ± 0.021	0.902 ± 0.025	$\mathbf{0.910 \pm 0.021}$
Pneumonia diagnosis ($n = 800$)	0.896 ± 0.019	$\mathbf{0.908 \pm 0.012}$	0.900 ± 0.014	0.907 ± 0.013
Alternative herbal medicine classification	0.857 ± 0.026	0.896 ± 0.023	0.916 ± 0.019	$\mathbf{0.918 \pm 0.017}$
Breast cancer subtyping	0.871 ± 0.014	0.887 ± 0.015	$\mathbf{0.889 \pm 0.011}$	0.888 ± 0.011

Table S2. Comparison of the four conformal prediction methods with single-model and compute-matched ensemble baselines on PneumoniaMNIST. Results are reported as mean ± standard deviation over 10 repeated experiments. Bootstrap-LR averages class probabilities from 100 logistic regression models trained on bootstrap samples of the complete sampled training set.

Training size	Method	Accuracy	Macro-F1
$n = 100$	LR	0.882 ± 0.019	0.866 ± 0.025
	Bootstrap-LR	0.886 ± 0.020	0.870 ± 0.025
	ICP	0.899 ± 0.020	0.894 ± 0.020
	BICP	0.898 ± 0.026	0.894 ± 0.026
	RICP	0.906 ± 0.017	0.901 ± 0.018
	OOB-ICP	**0.910 ± 0.015**	**0.905 ± 0.016**
$n = 200$	LR	0.889 ± 0.024	0.874 ± 0.030
	Bootstrap-LR	0.894 ± 0.024	0.879 ± 0.030
	ICP	0.896 ± 0.021	0.888 ± 0.024
	BICP	0.914 ± 0.027	0.907 ± 0.031
	RICP	0.910 ± 0.020	0.903 ± 0.023
	OOB-ICP	**0.918 ± 0.018**	**0.912 ± 0.020**
$n = 400$	LR	0.894 ± 0.026	0.879 ± 0.033
	Bootstrap-LR	0.904 ± 0.019	0.892 ± 0.022
	ICP	0.902 ± 0.028	0.892 ± 0.033
	BICP	0.916 ± 0.019	0.909 ± 0.021
	RICP	0.911 ± 0.021	0.902 ± 0.025
	OOB-ICP	**0.917 ± 0.018**	**0.910 ± 0.021**
$n = 800$	LR	0.898 ± 0.017	0.884 ± 0.021
	Bootstrap-LR	0.906 ± 0.015	0.894 ± 0.018
	ICP	0.906 ± 0.016	0.896 ± 0.019
	BICP	**0.917 ± 0.010**	**0.908 ± 0.012**
	RICP	0.910 ± 0.012	0.900 ± 0.014
	OOB-ICP	0.916 ± 0.011	0.907 ± 0.013

Table S3. Mean confusion-matrix entries for PneumoniaMNIST at $n = 200$ across 10 repeated experiments. Class 0 corresponds to normal and Class 1 corresponds to pneumonia. Values are mean numbers of test samples across repetitions.

Method	TN	FP	FN	TP
ICP	196.8	37.2	27.5	362.5
BICP	205.9	28.1	25.6	364.4
RICP	199.5	34.5	21.6	368.4
OOB-ICP	204.8	29.2	21.9	368.1

Table S4. Effect sizes, 95% confidence intervals, and exact p-values for the classification accuracy and macro-averaged F1-score comparisons with ICP. The effect size is defined as the mean paired performance difference $\Delta = M_{\text{method}} - M_{\text{ICP}}$, where M denotes the corresponding performance metric; positive values indicate higher performance than ICP. For the simulation and PneumoniaMNIST datasets, confidence intervals were calculated using the standard error of the paired differences. For alternative herbal medicine classification and breast cancer subtyping, confidence intervals were calculated using the Nadeau–Bengio corrected standard error. For each dataset and performance metric separately, the three prespecified comparisons (BICP versus ICP, RICP versus ICP, and OOB-ICP versus ICP) formed one multiplicity family, and p-values were adjusted within that family using the Holm procedure.

Dataset	Metric	Comparison	Mean Δ	95% CI	Raw P	Holm-adjusted P
Simulation	Accuracy	BICP vs ICP	-0.003	[-0.007, 0.002]	0.170	0.511
		RICP vs ICP	-0.001	[-0.004, 0.003]	0.722	1.000
		OOB-ICP vs ICP	-0.001	[-0.004, 0.003]	0.749	1.000
	Macro-F1	BICP vs ICP	-0.003	[-0.008, 0.001]	0.154	0.463
		RICP vs ICP	-0.001	[-0.005, 0.003]	0.625	1.000
		OOB-ICP vs ICP	-0.001	[-0.004, 0.003]	0.655	1.000
Pneumonia diagnosis	Accuracy	BICP vs ICP	0.018	[0.006, 0.029]	0.006	0.009
		RICP vs ICP	0.014	[0.006, 0.022]	0.004	0.009
		OOB-ICP vs ICP	0.022	[0.012, 0.031]	0.001	0.002
	Macro-F1	BICP vs ICP	0.019	[0.007, 0.032]	0.007	0.012
		RICP vs ICP	0.015	[0.005, 0.024]	0.006	0.012
		OOB-ICP vs ICP	0.024	[0.013, 0.035]	0.001	0.003
Alternative herbal medicine	Accuracy	BICP vs ICP	0.051	[0.026, 0.075]	0.001	0.003
		RICP vs ICP	0.072	[0.034, 0.109]	0.002	0.003
		OOB-ICP vs ICP	0.073	[0.037, 0.109]	0.001	0.003
	Macro-F1	BICP vs ICP	0.039	[0.019, 0.059]	0.002	0.003
		RICP vs ICP	0.060	[0.030, 0.090]	0.002	0.003
		OOB-ICP vs ICP	0.061	[0.034, 0.089]	0.001	0.002
Breast cancer subtyping	Accuracy	BICP vs ICP	0.017	[0.002, 0.032]	0.032	0.097
		RICP vs ICP	0.018	[-0.001, 0.038]	0.064	0.128
		OOB-ICP vs ICP	0.018	[-0.006, 0.041]	0.122	0.128
	Macro-F1	BICP vs ICP	0.016	[0.000, 0.032]	0.048	0.143
		RICP vs ICP	0.019	[-0.002, 0.040]	0.077	0.154
		OOB-ICP vs ICP	0.018	[-0.008, 0.043]	0.148	0.154

Table S5. Effect sizes and corrected 95% confidence intervals for the correct-versus-incorrect credibility comparisons. The effect size is defined as the mean paired difference $\Delta C = C_{\text{correct}} - C_{\text{incorrect}}$ across the 10 outer experimental repetitions. Confidence intervals were calculated using the Nadeau–Bengio corrected standard error, and p-values were adjusted using the Holm procedure.

Dataset	Method	Mean ΔC	Corrected 95% CI	Holm-adjusted P
Simulation	ICP	0.100	[0.027, 0.173]	0.012
	BICP	0.108	[0.039, 0.177]	0.012
	RICP	0.106	[0.064, 0.148]	0.001
	OOB-ICP	0.106	[0.069, 0.142]	< 0.001
Pneumonia diagnosis	ICP	0.300	[0.209, 0.392]	< 0.001
	BICP	0.268	[0.147, 0.388]	0.002
	RICP	0.293	[0.194, 0.392]	< 0.001
	OOB-ICP	0.277	[0.181, 0.374]	< 0.001
Alternative herbal medicine	ICP	0.391	[0.342, 0.439]	< 0.001
	BICP	0.268	[0.213, 0.324]	< 0.001
	RICP	0.314	[0.269, 0.359]	< 0.001
	OOB-ICP	0.314	[0.265, 0.363]	< 0.001
Breast cancer subtyping	ICP	0.292	[0.259, 0.324]	< 0.001
	BICP	0.278	[0.246, 0.309]	< 0.001
	RICP	0.295	[0.261, 0.328]	< 0.001
	OOB-ICP	0.295	[0.262, 0.329]	< 0.001

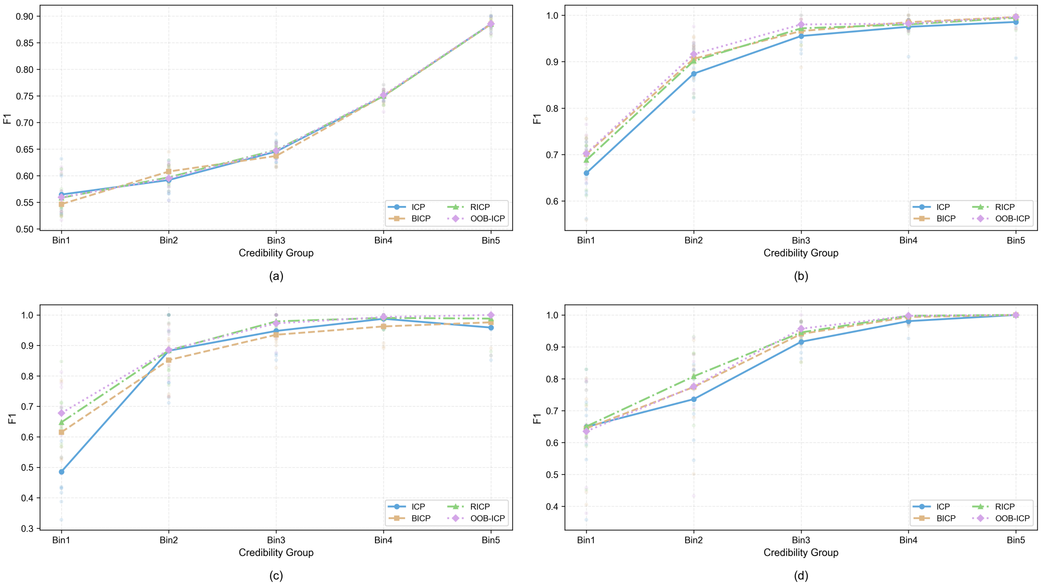

Figure S1. F1-score across five equal-frequency credibility groups. The trend is consistent with the accuracy-bin analysis, supporting the use of credibility as a reliability-ranking score.
